# Antibiosis interaction of *Staphylococccus aureus* on *Aspergillus fumigatus* assessed *in vitro* by mixed biofilm formation

**DOI:** 10.1186/s12866-015-0363-2

**Published:** 2015-02-15

**Authors:** Adrián Ramírez Granillo, María Gabriela Medina Canales, María Esther Sánchez Espíndola, María Angeles Martínez Rivera, Victor Manuel Bautista de Lucio, Aída Verónica Rodríguez Tovar

**Affiliations:** Laboratorio de Micología Médica, Depto. de Microbiología, Escuela Nacional de Ciencias Biológicas (ENCB), Instituto Politécnico Nacional (IPN). Carpio y Plan de Ayala s/n, Col. Casco de Santo Tomás, Del. Miguel Hidalgo, 11340 Mexico City, Mexico; Unidad de Microscopía ENCB, IPN, 11340 Mexico City, Mexico; Microbiology and Ocular Proteomics, Research Unit, Institute of Ophthalmology “Fundación de Asistencia Privada Conde de Valenciana”. Chimalpopoca 14, Col. Obrera, Del. Cuauhtémoc, 06800 Mexico City, Mexico

**Keywords:** Biofilm, Extracellular matrix, Antibiosis of *Staphylococcus aureus* against *Aspergillus fumigatus*, Fungus-bacteria interaction

## Abstract

**Background:**

Microorganisms of different species interact in several ecological niches, even causing infection. During the infectious process, a biofilm of single or multispecies can develop. *Aspergillus fumigatus* and *Staphyloccocus aureus* are etiologic agents that can cause infectious keratitis. We analyzed *in vitro* single *A. fumigatus* and *S. aureus*, and mixed *A. fumigatus-S. aureus* biofilms. Both isolates were from patients with infectious keratitis. Structure of the biofilms was analyzed through microscopic techniques including scanning electron microscopy (SEM), transmission electron microscopy (TEM), confocal, and fluorescence microscopy (CLSM) in mixed biofilm as compared with the single *A. fumigatus* biofilm.

**Results:**

To our knowledge, this is the first time that the structural characteristics of the mixed biofilm *A. fumigatus-A. fumigatus* were described and shown. *S. aureus* sharply inhibited the development of biofilm formed by *A. fumigatus*, regardless of the stage of biofilm formation and bacterial inoculum. Antibiosis effect of bacterium on fungus was as follows: scarce production of *A. fumigatus* biofilm; disorganized fungal structures; abortive hyphae; and limited hyphal growth; while conidia also were scarce, have modifications in their surface and presented lyses. Antagonist effect did not depend on bacterial concentration, which could probably be due to cell-cell contact interactions and release of bacterial products. In addition, we present images about the co-localization of polysaccharides (glucans, mannans, and chitin), and DNA that form the extracellular matrix (ECM). In contrast, single biofilms showed extremely organized structures: *A. fumigatus* showed abundant hyphal growth, hyphal anastomosis, and channels, as well as some conidia, and ECM. *S. aureus* showed microcolonies and cell-to-cell bridges and ECM.

**Conclusions:**

Herein we described the antibiosis relationship of *S. aureus* against *A. fumigatus* during *in vitro* biofilm formation, and report the composition of the ECM formed.

## Background

Antibiosis is an association between two microorganisms that is detrimental to at least one of them and that is caused by the release of metabolites or cell components [1]. Biofilm is a complex of cell populations associated with a biotic or abiotic surface and embedded into an extracellular matrix (ECM) of macromolecules with changes in their cellular physiology, representing a differential expression of genes [2]. Microorganisms are constantly interrelated in a natural and intimate mode when they colonize the surfaces to which they adhere. Mixed biofilms, among these, those built by fungus-bacterium interaction, are highly frequent. Formation of biofilm includes the following stages: adhesion; colonization; secretion of ECM; cell growth and expansion and, finally, dispersion [3,4]. Formation of a polymicrobial biofilm starts with the colonization of the surface by one of the constituting species, during which these planktonic species adhere to a surface and start the formation of structural scaffolds that serve as foundation for the biofilm. This sequential adhesion process is known as co-aggregation [5]. The mixed fungal-bacterial biofilm is formed by consortia of both microorganisms that are interacting. Contact and adhesion in fungal-bacterial interaction are fundamental events for the development of polymicrobial biofilm [6]. In some micro-consortia, the chemical composition of ECM is known (carbohydrate polymers, DNA and/or proteins), but others remain to be identified. The ECM envelops the microbial communities increasing surface adhesion; during infectious processes, this favors protection against the host, as well as resistance to drugs by the microorganisms [7].

Communication in polymicrobial ecosystems is accomplished by metabolites substances known as autoinducers; among some of these products are proteins, genetic material (DNA or RNA), and microbicide agents (bacteriocins, toxins). In symbiotic fungus-bacterium relations, *quorum sensing* molecules are the best studied, such as, for example, acyl-Homoserine-lactones (acyl-HSL) [8-10]. Autoinducer molecules exert several functions on these interactions such as chemotaxis and signalization, adhesion and antibiosis, among others [6,7].

Intrinsic interaction between fungi and bacteria has allowed for their co-evolution, enabling polymicrobial associations of synergism, antagonism, mutualism, among others. An example of synergism is described in *Candida albicans-Staphylococcus aureus* interaction, in which images obtained using confocal scanning laser microscopy, suggest that the yeast may provide and invasion strategy to staphylococci, due to carry over by candidal hyphae during their penetration through epithelial layers [11], another example of synergism is *C. albicans-Streptococcus gordonii* interaction; in mixed biofilm, hyphal development was enhanced and the formed biofilm consisted mainly of hyphae [12]. In both synergism interactions engage physical (adherence) and chemical (diffusible) signals that influence the development of biofilm communities. In the other hand, an antagonistic association was described during mixed biofilm were *Pseudomonas aureginosa* can attaches and kills filamentous *C. albicans* but neither attaches nor kills yeast-form cells [9], fungal signals can affect *Pseudomonas* gene regulation and motility and are likely to modified the ultrastructure of the mixed biofilm [10]. *P. aureginosa* has showed antibiosis to *A. fumigatus* by direct contact and secreted extracellular molecules [13]. Microscopic observations of bacteria–fungi interactions have showed that at least some antagonistic bacteria actively move towards and colonize the surface of fungal hyphae [14]. It is interesting that these interactions sometimes result in fungal apoptosis [15].

The study of polymicrobial biofilms has been increasing and, in the medical area, the role played by biofilms in co-infections has been associated with virulence factors, such as production and secretion of enzymes, proteins, and toxins, as well as adhesion processes, among others [16,17]. Hence, biofilm formation is also considered a determinant virulence factor for pathogenesis in the host [5,18]. Polymicrobial interactions are also reflected in eye diseases such as keratitis. During inflammation of the cornea, optimal conditions for the spread of the microorganisms in the eye are presented. Trauma to the ocular surface caused alterations in endothelium, edema, cellular infiltration among others, under these conditions and the colonization of microorganisms on abiotic surfaces, such as contact lenses, leads to the formation of biofilm into the eye [4]. The aim of this study was to analyze the ecological interactions between *S. aureus* on *A. fumigatus* during the formation *in vitro* of mixed biofilm: we found damage on fungal structures, display by SEM and TEM, and co-localization of structural components by CSLM. We showed evident images about structural changes of the ECM and qualitative characteristics of the fungus-bacteria interaction. Analysis of mixed biofilms formed by these microorganisms suggest an antibiosis effect of *S. aureus* on *A. fumigatus*, we described some of the mechanisms involved in this interaction that is so scarcely studied.

## Results

### Microbiological and molecular identification

*A. fumigatus* clinical isolate, in potato dextrose agar (PDA) medium for 5 days 37°C, developed the morphologic features of this species [19]. Molecular identification of *A. fumigatus* was performed and Basic Local Alignment Search Tool (BLAST) analysis of the nucleotide sequence of the ITS of (600 bp) revealed 100% homology with sequences reported for this fungus in the GenBank. The clinical *Staphylococcus aureus* isolate, was grown on BHI agar for 24 h at 37°C, and it exhibited the features of the species [20]. Analysis of the sequence of the 16S rDNA gene (1500 bp) of isolated *S. aureus* revealed 99% homology with the sequences reported for *S. aureus*.

### Biofilms formation and quantification

**Figure 1:**
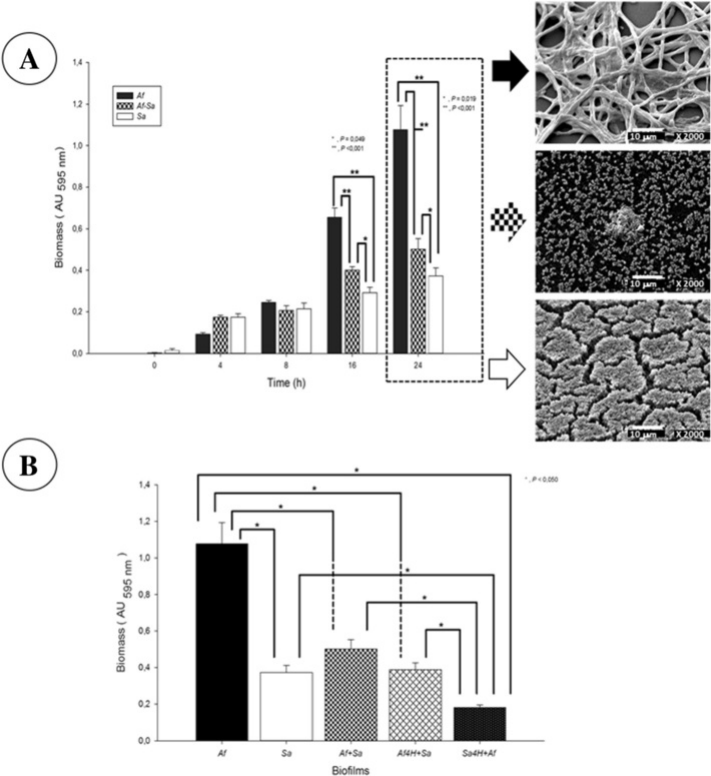
**Quantification and antagonism behavior of the single and mixed fungus-bacterium biofilm. A)** *Aspergillus fumigatus*, *Staphylococcus aureus,* and *A. fumigatus-S. aureus* biofilm quantification. Biofilm quantification at different incubation times (0, 4, 8, 16, and 24 h). Biofilm biomass was quantified indirectly by the crystal violet method (see materials and methods). In plotting these data, the mean of the absorbance and standard error of the mean are relative to n = 10 measures, comparisons between absorbance (relatives to biomass biofilms formation) were made and significant differences were determined by Student-Newman-Keuls test, performing multicomparison of procedures and the following are indicated: (*), *P* < 0.050; and; (**), *P* < 0.001. Values are representative of three experiments with ten replicates each one. At right, see *A. fumigatus, S. aureus and A. fumigatus-S. aureus* micrographic of biofilm structure at 24 h. **B)** Evaluation of the antagonism behavior of *S. aureus* on *Aspergillus fumigatus* during mixed biofilm formation. Fungus or bacteria inoculums were placed, as first colonizer, allowing adherence 4 h, and the missing microorganism was added. Used values are representative of three experiments with ten replicates each one; when plotting data, mean absorbance and Standard error of the mean are relative to *n* = 10. *Af* = *A. fumigatus; Sa = S. aureus; Af + Sa* = *A. fumigatus*-*S. aureus*; *Af4H + Sa* = *A. fumigatus* with 4 previous h of adhesion plus *S. aureus*; *Sa4H + A* = *S. aureus* with 4 previous hours of adhesion plus *A. fumigatus*. Significant differences were determined by Student-Newman-Keuls test, performing a multicomparison of procedures; significant differences are indicated as: (*), *P* < 0.050.

Biofilm formation was measured at 16 and 24 h, for single and mixed biofilms; however at 24 h the production was statistically higher (*p* < 0.001) (Figure 1A). In the *A. fumigatus-S. aureus* biofilm, the biomass detected (>0.2 AU) was significantly lower when compared with the single *A. fumigatus* biofilm (>0.4 AU) (Figure 1A). The antagonistic behavior of *S. aureus* on *A. fumigatus* during *in vitro* mixed biofilm formation is clear. In order to confirm the antagonistic behavior of *S. aureus* on *A. fumigatus* during *in vitro* formation of the mixed biofilm, we implemented fungus or bacterium inoculums, as first colonizer, allowing adherence during 4 h; subsequently, the missing microorganism was added, (see Material and Methods section). The high biofilm formation was observed in *Af* alone (>1.0 AU), whereas the *Sa* showed lower production of biofilm (<0.4 AU). In the mixed biofilms we observed that there was, a decrease in the biofilm formation in *Af + Sa* compared with *Af* but not with the *Sa*. When *S. aureus* was the first colonizer (*Sa*4H + *Af*) the biofilm formation was lower (<0.2 AU). When *A. fumigatus* was the first colonizer (*Af*4H + *Sa*; <0.4 AU), the biofilm formation was higher than *Sa*4H + *Af* but was lower compare to *Af* and *Af* + *Sa* treatments (Figure 1B).

### *A. fumigatus*, *S. aureus,* and *A. fumigatus-S. aureus* biofilm structure

**Figure 2:**
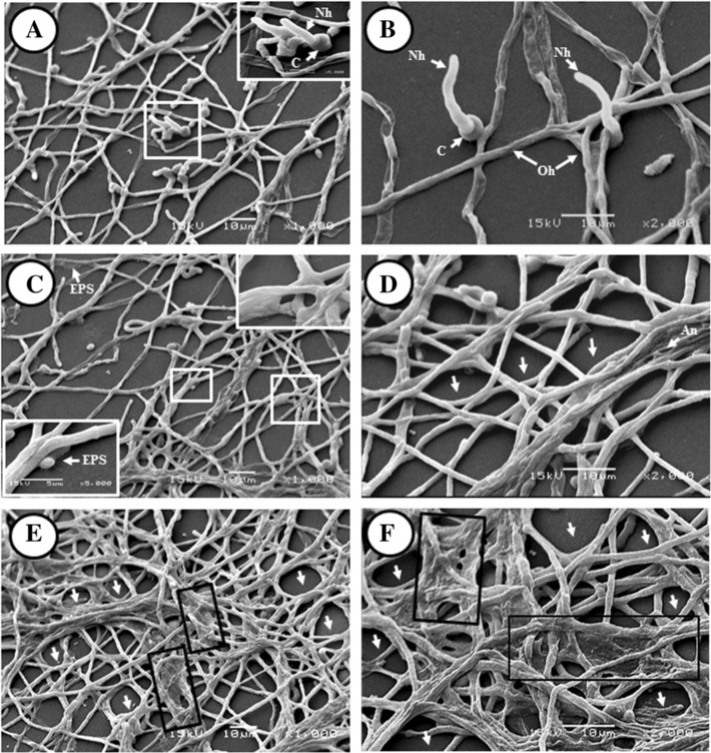
**Scanning electron microscopy (SEM) micrographic** ***in vitro A. fumigatus*** **biofilm.** During the biofilm formation process at 24 h, an asynchronous growth was observed. **A)** Conidia germination 1000X; **B)** new-generation hyphae on mature hyphae 2000X; **C)** exopolymeric substance production (EPS) and foundation of the biofilm 1000X; **D)** hyphae in anastomosis, channel formation and expansion of the hyphal network 2000X, **E-F)** increase of extracellular matrix (ECM) production and biofilm maturation 1000 and 2000X. White arrow channel formation; black box pointed ECM; C = conidia, ECM = extracellular matrix, EPS = exopolymeric susbstance, Nh = New generation hyphae, Oh = old hyphae.

Single *A. fumigatus* biofilm structural arrangements are shown with more details in Figure 2. The biofilm formation began with the adhesion of conidia at 4 h then the conidia initiated filamentation (Figure 2A). The sessile fungal cells (hypha and conidia) were surrounded by EPS enhancing adherence (Figures 2A-C). During the biofilm formation process, we observed asynchronous growth of conidia, which promoted the presence of young hyphae, standing out from the mature hyphae that worked as support for the biofilm (Figures 2A-B). The main characteristics of the fungal biofilm were observed during the maturation stage, represented by the abundant production of ECM, hyphal fusion (anastomosis), and the formation of aerial channels among the large mycelial networks (Figures 2D-F).

**Figure 3:**
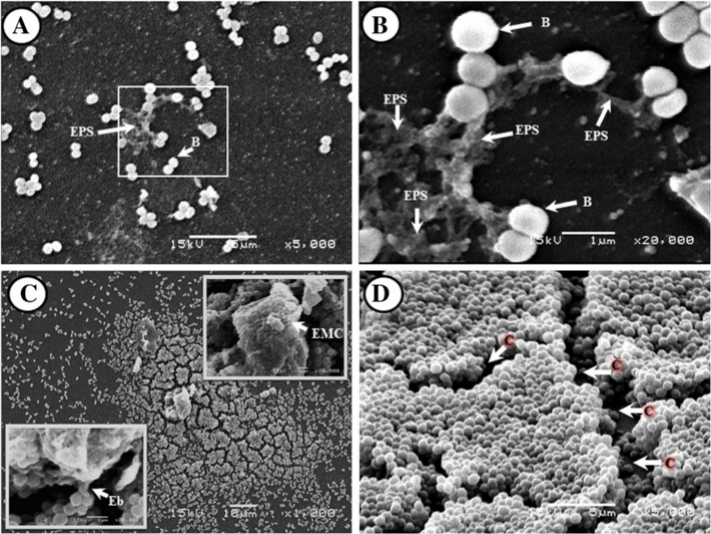
**Scanning electron microscopy (SEM) micrographic** ***in vitro S. aureus*** **biofilm. A)** aggregation and EPS production 5000X; **B)** aggregation and EPS production 20000X; **C)** expansion of the bacterial biofilm; polymeric bridge approaching closer (left inferior insert; 20000X); ECM in upper zone of the microcolony (upper right insert; 10000X); **D)** microcolonies of mature bacterial biofilm with ECM connecting bacteria and bacterial channels. C = channels, EPS = exopolymeric substances, B = bacteria, Eb = exopolymeric bridge, ECM = extracellular matrix.

*S. aureus* biofilm images through electron microscopy revealed the typical characteristics of bacterial biofilms. One of these was the organization in microcolonies with three-dimensional (3D) structures and rough topography (Figure 3). Abundant production of EPS was observed allowing the adhesion of cells to surface (Figure 3A, B). The ECM was produce at the mature biofilm and the formation of extensions known as polymeric bridges were present (Figures 3C). Finally the microcolonies present channels which have the capacity to move the fluids outside the microcolonies (Figure 3D).

**Figure 4:**
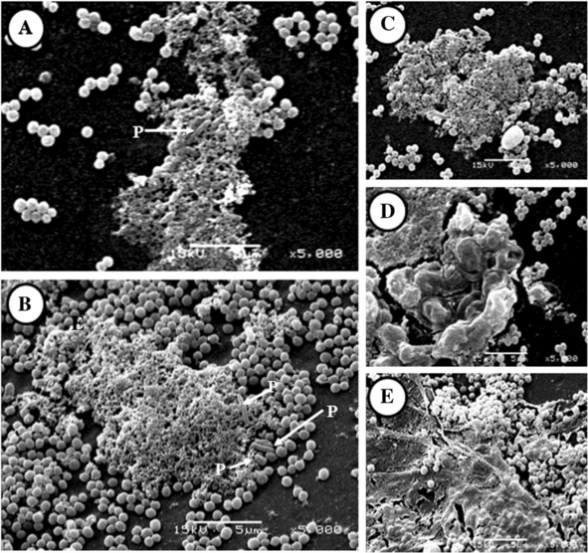
**Scanning electron microscopy (SEM) micrographic** ***in vitro A. fumigatus*** **-** ***S. aureus*** **mixed biofilm micrographic** ***.*** **A)** ECM surrounding fungal, bacterial and pleomorphic cells 5000X; **B)** ECM with pleomorphic cells in periphery 5000X; **C)** porous ECM 5000X; **D)** condensed ECM 5000X; **E)** film ECM 5000X. P = pleomorphic cells.

The mixed biofilm formed by *A. fumigatus*-*S. aureus* that depicts a completely different panorama was revealed by electron microscopy, when they were compared with single biofilms. These differences occurred in the ECM (texture and distribution) and in the cell (fungal and bacterial). ECM were demonstrated in some fragments with the appearance of porous ECM (Figure 4A-C) and condensed ECM (Figure 4D), whereas others covered the surface of large cocci groups like a film ECM (Figure 4E). Cellular modification were found the quantity of fungal cells was markedly reduced while the bacterial population was concentrated abundantly (without forming microcolonies) on the periphery of the different types of ECM. In addition, we observed the presence of cells with a morphology differing from the characteristic fungal or bacterial structures. The fungal cells resembled the very short and thin hyphae (abortive hyphae) that were found immersed in the EMC (Figure 4A-B). Regarding bacterial cells, morphologies neighboring the formed matrices were varied and abnormal with extracellular material secretion and evident cell division (Figure 4B).

### Fungus-bacterium interaction

**Figure 5:**
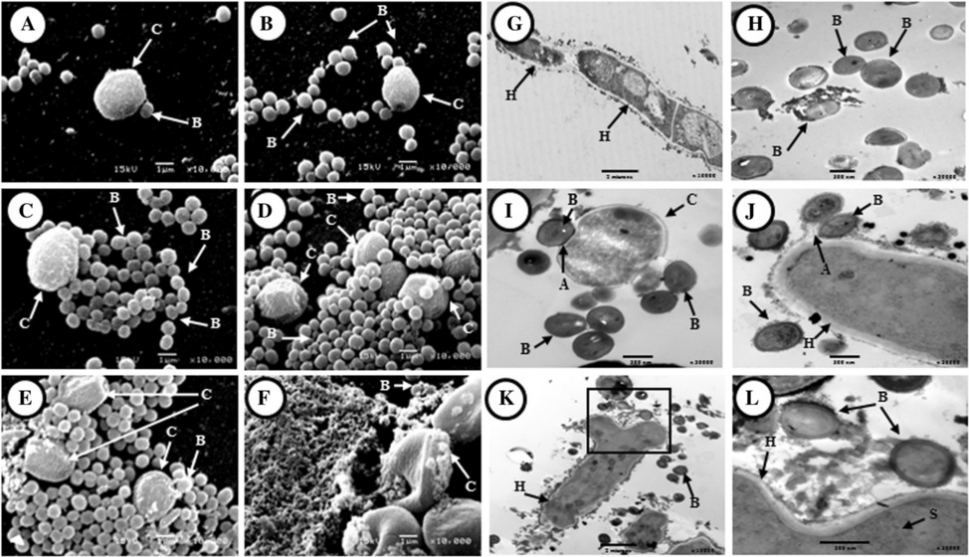
**Antagonistic effect of** ***Staphylococcus aureus*** **on** ***Aspergillus fumigatus*** **. A-F)** scanning electron microscopy (SEM) micrographic images 10000X. **A)** adhesion between conidia and bacterial cells; **B)** adherent bacterial on conidia; **C)** massive adhesion of cocci on conidia; **D)** morphological change of conidia; **E)** damaged surface of conidia, with cell lysis; **F)** cell lysis of *A. fumigatus* conidia, surface of conidia exhibits a smooth texture. **G-L)** Transmission electron microscopy (TEM) micrographic images. **G)** hypha of *A. fumigatus* on single biofilm 10000X; **H)** cocci of *S. aureus* on single biofilm 30000X; **I)** conidia surrounded by bacterial cells damaging the conidial wall cell 10000X (mixed biofilm); **J)** adhesion bacterial cells damaging the hypha 30000X (mixed biofilm); **K)** hypha with invagination at the apical zone, lysis of the hypha is observed on the end pole 10000X (mixed biofilm); **L)** deformation of the hyphal apical zone at a higher magnification 30000X (mixed biofilm). B = bacteria, H = hypha, A = adhesion, C = conidia, S = spitzenkörper.

SEM revealed evident structural alterations in the fungus-bacterium interaction, particularly in fungal structures, although was observed in bacteria also. Adhesion of *S. aureus* to *A. fumigatus* structures was observed (Figure 5A); bacterial adhesion increased (Figure 5A) and the conidia were enveloped by cocci agglomerations (Figures 5B-E). Morphological changes in conidia texture (from rough and irregular to a loose structure) were noted; additionally, there were alterations on the surface of conidia (structural distortion), damage to the poles, wall erosion, and pore formation, and limited hyphae development (Figures 5C-F). Finally, this interaction gave rise to fungal lyses with the release of cytoplasmic material to which bacteria bound (Figure 5F). As a final point, a marked decrease in biofilm formation was observed in each microorganism.

The TEM revealed, as shown in Figure 5I-L, that there was a direct interaction between cocci and fungus via extracellular material comparing with the single biofilm of *A. fumigatus* and *S. aureus* (Figure 5G-H). It was observed that the conidia and hyphae were surrounded by cocci and the conidial wall was damaged (Figures 5I-J); there were morphological alterations in fungal structures at the hypha wall or membrane level, and lyses of conidia. Figures 5K and L shown the interaction of *S. aureus* with the apical growth zone of the *A. fumigatus* hypha (It was branching) a close up (Figure 5L) show the interaction via extracellular material.

### Structural composition of ECM by CLSM

Images obtained by SEM and TEM, described previously, for single and mixed biofilms exhibited evident alterations; therefore we proceeded to analyze the composition and distribution of some polymers using fluorochromes in single fungus biofilm and in a mixed biofilm. Biofilms formations were those described previously and the following fluorochromes were utilized: calcofluor white, green halo (chitin); FUN®1, red halo localized inside the hypha (metabolic activity); conA, yellow halo (glucose and mannose residues); and fluorescent dye 6-diaminidine 2-phenylindole (DAPI), blue halo and propidium iodide (PI), red halo (nucleic acids).

**Figure 6:**
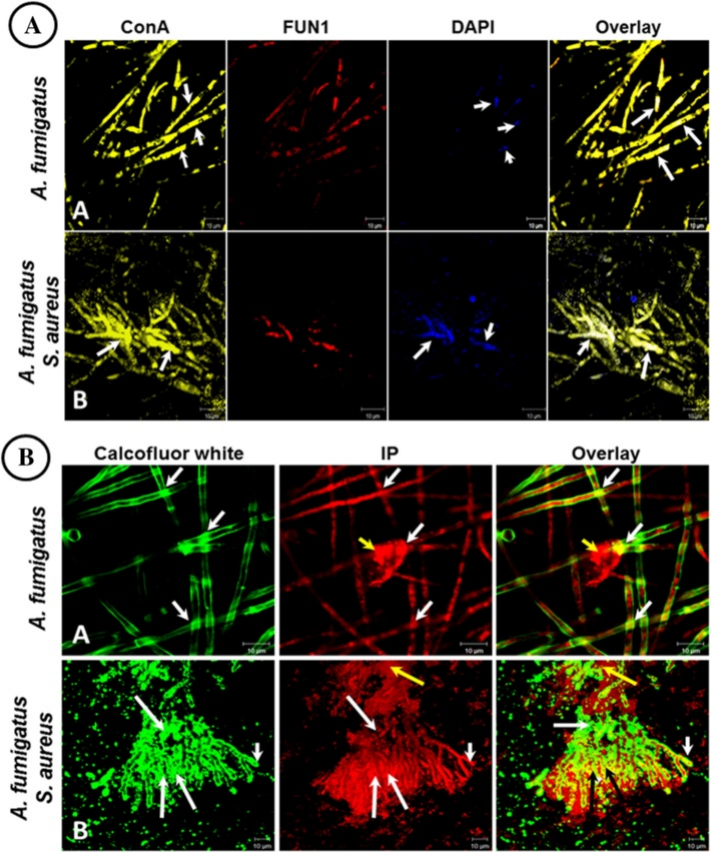
**Structural composition of ECM in single** ***Aspergillus fumigatus*** **and mixed** ***A. fumigatus-Staphylococcus aureus in vitro*** **biofilms by CLSM. A)** co-localization of mannose and glucose/DNA, fluorochromes used are indicated on upper part. Overlapping images show white or intense yellow halos, evidencing molecular co-localization. *A. fumigatus* biofilm (100X) and mixed *A. fumigatus*-*S. aureus* biofilm (63X). **B)** co-localization of chitin/DNA, the fluorochrome used is indicated on upper part. Overlapping images show intense yellow halos, evidencing co-existence of chitin and DNA. White arrowheads indicate co-localization of macromolecules; yellow arrowheads indicate ECM; white arrow corresponds to hyphae with nule or scarce metabolic activity without concanavalin A (conA) signal.

Co-localization among polysaccharides was revealed by overlapping of the signals emitted by the fluorochromes when they bounded to mannose/glucose and DNA, was observed in both biofilms as a white or intense yellow halo on the overlapping site of chitin and DNA (conA, yellow halo; DAPI, blue halo) (Figure 6A, white arrowhead). In the mixed *A. fumigatus*-*S. aureus* biofilm, fluorescence was greater in this molecular disposition in abortive hyphae (Figure 6A-B, white arrowhead). This finding suggests an excessive production of ECM by each microorganism. Likewise, co-localization of chitin and DNA was confirmed in the biofilms with the mixture of calcofluor white (green color) and PI (red color) (Figure 6B). The presence of intense yellow halos manifested overlapping of the fluorescence emitted by each molecule. Additionally, in the single *A. fumigatus* biofilm, mucoid material formation was observed around the hypha (Figure 6B-B, yellow arrowhead) and was detected only with PI, suggesting that EMC was also formed by DNA. In the mixed biofilm, abnormal hyphae surrounded by a large bacterial population were observed, revealing the co-existence of DNA and chitin (Figure 6B-B, white arrowhead).

### Antibiosis of *S. aureus* on *A. fumigatus in* mixed biofilm formation

**Figure 7:**
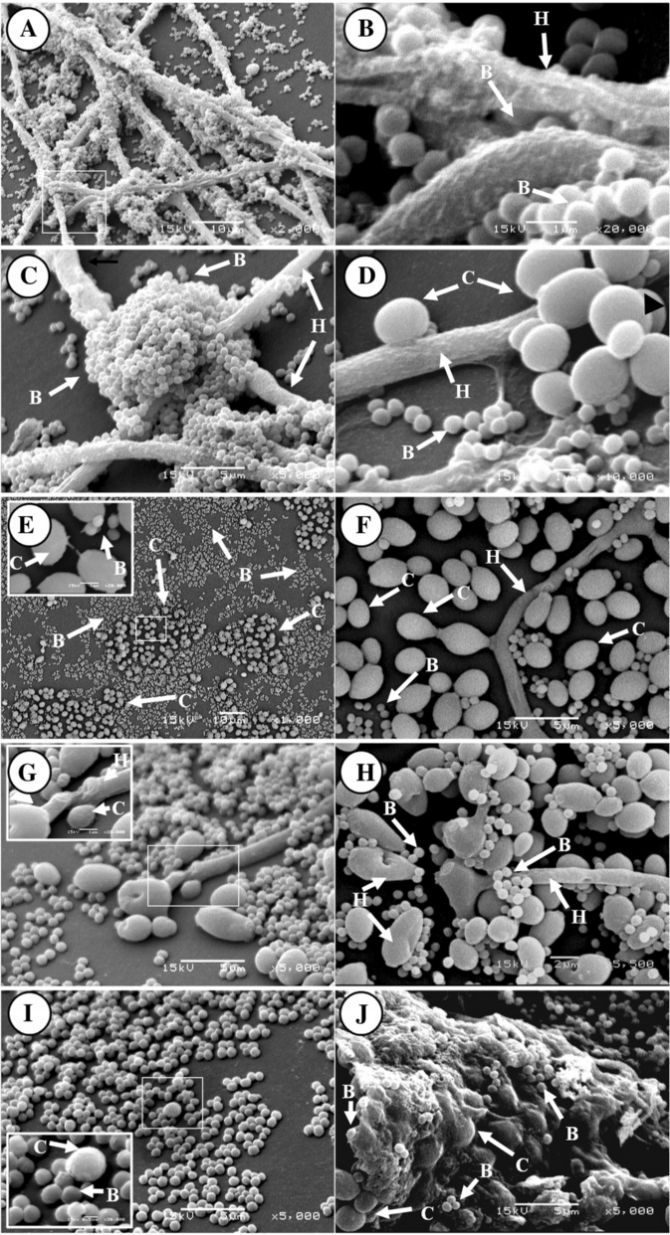
**Antibiosis in mixed** ***Aspergillus fumigatus*** **-** ***S. aureus*** **biofilm by SEM.** Assessment of concentration of the *Staphylococcus aureus* inoculum in the development of the mixed *Aspergillus fumigatus-S. aureus* biofilm*.* **A-D)** *A. fumigatus*-*S. aureus* biofilm, inoculum of 1 × 10^3^ bacteria/mL: **A)** bacterial aggregation on extended hyphae 2000X; **B)** close-up of Figure A1, note bacteria within anastomosed hyphae 20000X; **C)** bacterial clustering around hyphae with altered morphology 5000X; **D)** bacterial cells on ECM, in contact with ECM of hyphae, neighboring a conidial cluster 10000X. **E-J)** mixed *A. fumigatus*-*S. aureus* biofilm with an inoculum of 1 × 10^7^ bacteria/mL: **E)** conidia surrounded by abundant *S. aureus* populations, conidia with prolongations 1000X (upper left insert 20000X); F) active conidation 5000X; **G)** group of atrophied hyphae with conidia surrounded by cocci, remainder of fragmented hypha 5000X (upper left insert, 20000X); **H)** bacterial damage on hypha in vertices 5500X; **I)** bacterial population enveloping the fungal structure; insert in lower left part shows damaged conidia with morphological change 5000X (upper left insert, 20000X); **J)** mixed *A. fumigatus*-*S. aureus* biofilm 5000X. Biofilms developed on 12-well polystyrene plates incubated 24 h with RPMI medium at 37°C. White box insert-higher magnification detail. B = bacteria, C = conidia, H = hypha.

The antibiosis of *S. aureus* on *A. fumigatus* mixed biofilm was observed through SEM. In Figures 7A-D. We observed that *A. fumigatus* was a decrease in the biofilm formation and the fungal growth was inhibited even at low *S. aureus* concentrations using 1 × 10^3^ bacteria/mL as inoculum (data not shown). In these conditions fungal filamentation was defective (Figure 7A) and hyphae presented damage in their cellular wall (Figure 7B); conidiation in this biofilm was moderate (Figures 7C-D); EPS favors the fungus-bacterium interaction, and both microorganisms could be participating in the antagonist phenomenon exerted by *S. aureus* on *A. fumigatus* (Figure 7D).

In figures 7E-J, the mixed *A. fumigatus-S. aureus* biofilm with 1 × 10^7^ bacteria/mL, a minimal hyphal development occurred as compared with the previous model (Figure 7A-D). The amount of detected hyphae was scarce and they depicted greater damage; we observed morphologically atrophied structures, which we have denominated abortive hyphae (Figures 7G-H). Some conidia-producing hyphae were also observed; however conidia were enveloped by bacterial cells; filamentation was limited; thus, mycelium formation was restricted (Figures 7E-F); in addition, important morphological changes were observed in conidia, such as wall damage and size variations (Figure 7I). Conidia in the germination process were scarce (Figure 7G). Likewise, hyphae ramification was restricted (Figure 7H). We observed some conidia with prolongations which, interact with other cells (Figure 7E, upper left insert). These cell prolongations were more evident when the bacterium interacted with conidia in the mixed biofilm (Figure 7E). Finally, the adhesion, integration, and interaction of both microorganisms enabled the formation of the mixed biofilm. Although the biofilm was only in scarce zones (Figure 7J) where the fungal structures and bacteria were embedded in the ECM formed in that niche, despite their antagonistic interaction.

## Discussion

Microbial consortia comprise a large variety of species such as fungi and bacteria, among others. In some cases, they are able to generate infections that disseminate by adhering to host cells, forming groups, colonizing, and producing an ECM, composed of exopolymers and, eventually, forming biofilms [3,4]. An example can be provided by polymicrobial keratitis caused by a fungus-bacterium infection [4,21]. In this study, we report, to our knowledge for the first time, an antibiosis effect of *S. aureus* on *A. fumigatus* on mixed biofilm assessment by SEM, TEM and CLSM images. The antagonist behavior of the interaction was established amoung clinical isolates from patients with microbial keratitis, *A. fumigatus,* and *S. aureus,* during *in vitro* biofilm formation on abiotic surfaces. *S. aureus* exerted constant inhibition on the mixed biofilm formed by *A. fumigatus-S. aureus*, independently of the stage of biofilm formation as well as from the bacterial inocula, damage to fungal structures that included lyses of conidia; development of abortive hyphae and severe alterations in the structure and amount of the ECM formed were evidenced.

An assessment of alterations caused by *S. aureus* to *A. fumigatus* was performed by comparing single biofilms with those observed in the mixed biofilm. Broadly speaking, *A. fumigatus* biofilm was greater than *S. aureus* biofilm and mixed biofilm (Figure 1A). This effect could be related with three aspects that differentiate a bacterial biofilm from a fungal one: type of growth; metabolic activity, and variation in cell morphology [22].

*A. fumigatus* biofilm was built by structures that revealed asynchronous fungal growth, which enables new-generation hyphae to continue germinating from conidia deriving from mature hyphae (Figures 2A, B). This process allows the formation of channels, which are specialized structures for water and nutrient transport, as well as for toxic metabolite removal (Figure 2D-F). These structures have also been reported in biofilm of *A. niger* on polyester surfaces [23]. Another important feature of fungal biofilm is the secretion of extracellular polymeric substances (EPS), which are detected in the micrograph (Figure 2C) as a mucoid substance covering fungal structures. EPS production increases and closes the lumen of some channels and the interstitial spaces among them, generating the ECM formed by *A. fumigatus* (Figure 2E, F) [22,23]. The ECM helps to fuse the hyphal skeleton that will support the tridimensional (3D) structures. Hyphae presented anastomosis and the ECM enveloped them, thickening the hyphal support (Figure 2E, F). Similar structures were observed by Seidler *et al*. in *A. fumigatus* biofilms formed *in vitro* [24].

In the *S. aureus* biofilm, the formation of compact microcolonies with ECM release was evident (Figure 3C, D); additionally, there are links or ties among neighboring groups of bacteria by means of the formation of polymeric bridges (Figure 3C, left insert), also described by Characklis and Cooksey [25], which can adhere to the substrate through ECM-surface interactions established by electrostatic and Van der Waals forces, among others [3,25-27].

Bacterial antagonism on *A. fumigatus* and bacterial alterations were evidenced by the SEM images, which illustrates reduced germination of the fungus in the mixed biofilm; fungal structures are considerably lower in the *S. aureus* population*.* Also, the bacterium responds to the fungus-bacterium interaction as revealed by the limited formation of microcolonies. In addition, we observed the ECM of diverse topographies and textures that were completely different from those produced in each single biofilm (Figures 4C-E). In some cases, it was possible to evidence pleomorphic cells both embedded in the matrices and neighboring the periphery of these amorphous structures (Figures 4A, B). The fungal or bacterial origin of these pleomorphic cells must be studied further. Studies performed by Ramage et al. [28]. revealed that the *C. albicans* biofilm modifies its morphological structure when subjected to stressing environmental conditions (e. g. farnesol), in which short and atypical hyphal formation is observed, as well as inhibition of filamentation [28]. In this study, a similar event in the mixed *A. fumigatus-S. aureus* biofilm was observed, in which hyphae with atypical morphology were found, which we denominated abortive hyphae [29,30], due to their small size and thickness (Figure 4A, B). We suggest that the morphological effect is not only related with the fungus-bacterium contact, but is also a result of the effect caused by some unknown metabolite produced by *S. aureus*. In addition, the effect was also observed in bacterial cells in the mixed biofilm; previous studies demonstrated electron microscopy images of heterogeneous bacterial cells, in shape and size, such as the small colony variants (SCV) observed when these are isolated from patients subjected to lengthy antibiotic treatment, favoring stress conditions. These authors also reported a large secretion of extracellular material and accelerated cell division [31]. These bacterial morphological changes are similar to those observed in our study in the mixed biofilm. The *A. fumigatus*-*S. aureus* interaction in the mixed biofilm suggests that the antagonistic bacterial activity exerted on the fungus could be attributed to an extracellular biotrophic phenomenon accompanied by a necrotrophic-type interaction [14]. In this possible antibiosis effect, *S. aureus* was adhered to conidia, and probably caused chemotaxis and the attraction of other bacterial cells. Conidial alterations in shape and surface are clear and, also observed deformations, aside from a loose texture, ending in lysis of the conidium; also observed are bacteria interacting with the released material (Figure 5A-F).

Reports about *Cryptococcus neoformans-S. aureus* co-cultures by Ikeda et al. [15], utilizing SEM, the authors observed that *S. aureus* also possesses high affinity for *C. neoformans* structures, which are eventually lysed. Additionally, the characteristics of *C. neoformans* blastoconidia are very similar, to the damage caused by *S. aureus* on the conidia of *A. fumigatus*; this action of the bacterium could be related with damage on the wall of the conidia, which is rich in polysaccharides and which might be used as substrate (as described for the *C. neoformans* capsule). Similarly, in the *A. fumigatus*-*S. aureus* mixed biofilm, we observed that bacterial adhesion is always present for this antagonistic effect (Figure 5I-L). Ikeda et al. [15], deduced that the death of *C. neoformans* by *S. aureus*, was caused by adhesion to polysaccharides with mannose residues, in combination with the activity of proteins such as the triosephosphate isomerase enzyme that interacts with fungal carbohydrates. The ECM of *S. aureus* contains a large variety of molecules that favor adhesion, such as polysaccharide intercellular adhesion (PIA), polymeric N-acetyl-glucosamine (PNAG), and microbial surface components recognizing adhesive matrix molecules (MSCRAMM), which are constituents of the microbial surface [32,33]. It is possible that, likewise, the interaction of this group of molecules present in bacterial ECM unchains a series of events resulting in an antagonistic effect on the fungus, in which specific receptors, participate allowing for an irreversible-type of adhesion [32].

The antagonisms of *S. aureus* can also observed on *A. fumigatus* in the TEM images. In Figure 5K and L, an electro-dense material can be observed between the bacterium and the adjacent conidia; this material possibly corresponds to metabolites released by bacteria and/or fungi, which could be causing the apical deformation of hyphae, an area of high enzymatic activity [34]. In this apical zone of hyphae (spitzenkörper) that is branching, and appear short and thick, it is possible an abortive hypha. These events could explain the fungal growth limitation exerted by *S. aureus*. Brandl et al. [35], studied a mixed biofilm formed by *Aspergillus niger*-*Salmonella typhimurium,* which showed a similar adhesion effect with high affinity for the apical zone of hyphae, covering it in a few hours. Furthermore, on the opposite pole, lysis of the hypha is observed (Figure 5K) [35-37], an effect that is also caused in extracellular necrotrophism [14]. Based on these results, we suggest the possibility that a product from *S. aureus* can be the cause of the death of *A. fumigatus.* Earlier, we commented that *S. aureus* employs enzymes that produce metabolic disequilibrium in *C. neoformans*, and it is possible that these bacterial components present enzymatic activity [15]. However, it is possible that they could be exotoxins, due to that *S. aureus* secreted wide range of molecules with diverse activities [38,39], and similar antagonisms were observed on the mixed biofilm of *A. fumigatus-P. aeruginosa*, in which was inferred that the effect was caused by direct contact and diffusible extracellular molecules of bacterial origin [13].

In order to assess the antagonistic effect of *S. aureus* on *A. fumigatus*, during the adhesion stage in the mixed biofilm, we performed assays alternating the primary colonizer, changing the inoculum concentration and the adhesion time. Mixed biofilm production is determined by the adhesion of the primary colonizer (Figure 1B- *Sa4H* + *Af*). This antagonistic bacterial activity by inhibition of the development of the mixed biofilm can be compared with the work, *in vivo*, of Mohan et al. [40], in which the authors simulated a corneal infection by inoculating an *A. fumigatus* and *S. aureus* suspension into a rabbit’s eye. They observed that the tissular injury is smaller on first inoculating the bacterium followed by *A. fumigatus*, as compared with the ulcers produced when inoculating the fungus individually. The results of this *in vivo* model resemble our *in vitro* biofilm, in which *A. fumigatus* produced a larger scale biofilm.

During adherence and antibiosis in the fungal-bacterial interaction, fungal structures of *A. fumigatus* showed some relevant biological event. Conidia include prolongations that possibly serve for cellular recognition (Figure 7E, upper left insert). These cell prolongations were more evident when the bacterium interacted with the conidia. This event has been reported in studies on a single *A. fumigatus* biofilm by Shopova et al. [41], who described it in *A. fumigatus* strains isolated from the lungs of patients with cystic fibrosis, with an environment rich in mucoid material abounds. The authors mention that there is a large concentration of free DNA deriving from a process caused by neutrophils and that is termed NETosis, a type of cell death in which the DNA of neutrophils is expelled in large filaments that form net-like structures known as neutrophil extracellular traps (NETS) [42]. These, in turn, contain antimicrobial peptides that help eliminate bacteria. It is possible that the fungus alters *S. aureus* biofilm formation, avoiding the characteristic bacterial organization. On the other hand, Shopova et al. [41] suggest that the extracellular DNA (eDNA) provides support to conidia on the adhesion surface; hence, this free DNA is part of the ECM that envelopes the fungal biofilm, together with hyphal networks that appear to be similar neutrophils’ NETS.

Assays to determine the structure and chemical composition of single *A. fumigatus* and mixed biofilms, using specific fluorochromes for each component, revealed a molecular co-localization phenomenon among polysaccharides (chitin and glucose/mannose), as well as of DNA-polysaccharides. Polysaccharides comprise part of the ECM released by the fungus (chitin, α-1, 3 glucans and galactomannans) [22,43]. Overlapping of DNA and polysaccharides in anastomosis sites or where hyphae crosslink, suggest that this co-existence provides rigidity and support, particularly in the biofilm bed during the initial formation stages. This organization was also described in a single fungal biofilm by Shopova et al. [41], With regard to the mixed *A. fumigatus*-*S. aureus* biofilm, this is, to our knowledge, the first description of this phenomenon of molecular co-localization. In addition to this effect, we evidenced fluorescent emission surrounding the hyphae, suggesting that this could be material synthesized by the fungus. The FUN1 marker revealed that the metabolic activity is determinant for the production of EPS; hyphae with low or null metabolic activity did not show fluorescence with conA (Figure 6A, white arrow). In the *A. fumigatus* biofilm (Figure 6B; yellow arrowhead), EPS zones marked with PI exhibited fluorescence around hyphae; this signal, overlapping in structures marked with calcofluor white, emitted yellow-colored fluorescence due to co-localization of molecules. These results suggest that the mixed biofilm presents, in its ECM, exopolysaccharides and DNA deriving from the fungus. According to Shopova et al. [41], DNA confers a more solid and resistant structural organization when it is co-localized with polysaccharides. It has been suggested that the DNA derives from fungal cells due to the secretion of chitinases by *A. fumigatus* favoring its release in early stages of biofilm formation [37]. This is important for the establishment of hyphal foundations embedded in the ECM. The fluorescence of the three fluorochrome mixtures emitted by the mixed biofilm is greater than that emitted by the single *A. fumigatus* biofilm, which suggests it is related with DNA-polysaccharide co-localization (Figure 6). This effect also suggests that the antibiosis of the fungus-bacterium interaction is enhanced, in which the ECM produced favors the fungus death by *S. aureus*, damaging the fungal wall and releasing DNA, events extensively shown in this study [37].

## Conclusion

In conclusion, this is, to our knowledge, the first report showing an antagonistic effect induced by *S. aureus* on the mixed *A. fumigatus-S. aureus* biofilm in fungal development. Fungal conidiation, filamentation and subsequent biofilm formation of *A. fumigatus* is inhibited by *S. aureus* during the mixed biofilm formation. The bacterium significantly limits fungal growth, probably through cell-cell contact and by means of the synthesis of bacterial products. In addition, we observed parts of the ECM composition formed during the fungal-bacterial interaction, for the first time, accompanied by the co-localization phenomena in the ECM of some molecules that, up to now, had only been reported in single biofilms of these microorganisms. Likewise, we also determined that inhibition of *A. fumigatus* biofilm formation by the bacterium is greater when the latter adheres first to the surface, even with low bacterial inocula, adhesion were critical in fungus-bacterium interaction. This event can be related with clinical observations by ophthalmologists in cases of infectious keratitis, who found better clinical evolution when the infection is mixed (fungus-bacterium), as well as with other published works. Therefore, these findings could be employed as therapeutic alternatives and provide clinical information for the study of eye infection treatments.

## Methods

### Biological material

*A. fumigatus* and *S. aureus* were isolated from patients with keratitis, these patients were seen at the *Instituto de Oftalmología “Fundación Conde de Valenciana”*in Mexico City. The current investigation has been performed with the approval of the *Institute of Ophtalmology “Fundación Conde de Valenciana IAP”* Science and Bioethics Research Committee, with reference number CEI-2013-08/02. The *A. fumigatus* isolate was grown in potato dextrose agar (PDA) (BD Bioxon, Mexico) medium at 37°C for 5 days. The *S. aureus* isolate was seeded by cross-streaking on Brain heart infusion (BHI) agar (BD Bioxon, México) plates at 37°C and, incubated for 24 h.

### Microbiological and molecular identification

*A. fumigatus* and *S. aureus* clinical isolates were identified by microbial methods, colony and microscopic morphology as well as biochemical profiles [19,20]. For molecular identification, genomic DNA was obtained by the Allers and Lichten method [44] for both isolates. The fragment ITS1-5.8S rDNA-ITS2 was used for the identification of *A. fumigatus*, following the amplification protocol described by Gardes and Bruns [45], and for the identification of *S. aureus,* the sequence of the *16S* rDNA gene was amplified using the universal primers fD1 and rD1 following the protocol described by Weisburg et al. [46]. The obtained amplicon was sequenced at the Molecular Biochemistry Laboratory, UBIPRO, FES Iztacala, UNAM (Mexico City, Mexico). A comparative analysis of the nucleotide sequences using the Basic Local Alignment Search Tool (BLAST) program (http://www.ncbi.nlm.nih.gov/) was also carried out.

### Biofilm formation and quantification

Single biofilms of *A. fumigatus* and *S. aureus*, and mixed *A. fumigatus-S. aureus* biofilms were formed on 96-well flat bottom polystyrene plates (Nunc Roskilde, Denmark). For the *A. fumigatus* biofilm conidia harvested of the aerial static culture was used, according to the method described by Mowat, et al. [13], and these were suspended in RPMI medium added to glucose at 2%. Preliminary assays showed that the best biofilm was observed at 1 × 10^5^conidia/mL for *A. fumigatus* and 1 × 10^8^ bacteria/mL for *S. aureus*. Biofilms were formed by adding 100 μl of conidial and/or bacterial suspension in RPMI supplemented to glucose 2% to each well. The plate were incubated at 37°C for 4 h (adherence stage), the supernatant was eliminated to remove the non-adherent cells, and 200 μL of freshly RPMI medium was added. Plates were then incubated for 0, 4, 8, 16, and 24 h. After that, the medium was eliminated and the wells were washed with 200 μL phosphate buffered saline (PBS). Biofilm biomass quantification was performed indirectly through the method described by Christensen et al. [47], modified by Peeters et al. [48] and a minor modification was made by Ramírez-Granillo [49], which used crystal violet, at 0.005% (final concentration). The dye excess was removed with distilled water and left to air dry. Finally, the dye bound to the biofilm was solubilized with 200 μL of acetic acid (J.T. Baker, Phillipsburg, NJ, USA) at 33% (v/v) for 15 min, the solution was transferred to a clean 96-well microtitre plate, and read at 595 nm in a microplate reader (Multiskan Ascent Thermo Labsystems; AIE, Waltham, MA,USA). The OD values are proportional to the quantity of biofilm biomass. Ten different biofilm for a same model, were used and the experiment performed on three separate occasions.

Additional assays in order to analyze the antagonistic behavior *in vitro* of *S. aureus* on *A. fumigatus*. The mixed biofilm was developed as described previously with the following modifications: the fungus and the bacterium were inoculated independently and incubated for 4 h; after that, the missing microorganism for the fungus-bacterium interaction was inoculated and incubated at 37°C during 24 h.

### Statistical analysis

Absorbance values of the single (*A. fumigatus* or *S. aureus*) and mixed (*A. fumigatus* - *S. aureus*) biofilms were compared using a two-tailed analysis of variance (ANOVA); Student-Newman-Keuls test was used to determine significant differences employing the SigmaPlot ver. 12.0 software (Systat Software Inc., San Jose, CA, USA).

### Analysis of biofilms structure by scanning and transmission electron microscopy (SEM and TEM)

Single and mixed biofilms for electron microscopy were developed as described in the biofilm formation section above, but in 12-well polystyrene plates at 37°C during 24 h (Santa Cruz Biotechnology, Santa Cruz, CA, USA) were used for this experiment. For SEM, samples were processed as described by Bozzola and Russell [50] and by Vázquez-Nin and Echeverría [51]. Briefly, the biofilms were washed with PBS and fixed with 2% glutaraldehyde (Electron Microscopy Sciences®, Washington PA.) for 2 hours. Then the biofilms were post-fixed with 1% osmium tetroxide (Electron Microscopy Sciences®, Washington PA.) for 2 hours. The bottom of the 12-well polystyrene plates was cut with a hot punch and the intact biofilm was obtained. The samples were dehydrated with ethanol at 10, 20, 30, 40, 50, 60, 70, 80 and 90% for 10 minutes and absolute alcohol for 20 minutes. Then the biofilms were put into a critical point dryer and were coated with ionized gold for 400 seconds at 15000 KV and 10 μA. The samples were observed in a scanning electron microscope (JEOL, Tokyo, Japan). Biofilm samples for TEM were processed as for SEM but they were then embebbed into resin and left to polymerize overnight. We performed semi-fine sections with a Leica Ultracut UCT (Wetzlar, Germany) microtome, and contrasted with lead and uranyl solutions. Finally, specimens were mounted on slides for observations under the microscope (JEOL Tokyo, Japan).

### Structural composition of ECM by confocal laser scanning microscopy (CLSM)

Single and mixed biofilms were developed as described above on 12-well polystyrene plates covered with a sterile coverslip (Velab, Mexico City, Mexico).

Coverslips were recovered and placed in contact with a mixture of fluorochromes. The following fluorochromes were applied: calcofluor white 1 g/L (Sigma-Aldrich St. Louis, MO, USA) for chitin; 10 mM FUN®1 (Life Technologies, Gaithersburg MD, USA) for metabolic activity; 1 mg/mL concanvalin A (conA) (Sigma-Aldrich St. Louis, MO, USA) for glucose and mannose residues; and 1.5 μg/mL DAPI (Vector Laboratories, CA, USA) and propidium iodide (PI) 10 mg/mL (AbD Serotec, Raleigh, NC, USA) for nucleic acids. Samples were observed under CLSM (Carl Zeiss, Germany) with filters: 480-530 nm (FUN®1), 360-460 nm (DAPI), 543-560 nm (PI), 355-433 nm (calcofluor white), and 495-519 (conA). Images were processed with Zeiss LSM Image Brower ver. 4.0 software (Carl Zeiss, Germany).

### Antibiosis of *S. aureus* on *A. fumigatus* in mixed biofilm

We performed additional assays in order to analyze the antibiosis behavior of *S. aureus* on *A. fumigatus*. The mixed biofilm was developed as described in the biofilm formation section above, with the following modifications: the bacterium inocula were increase in concentration since 1 × 10^3^ until 1 × 10^7^ bacteria/mL. The fungal inoculum was constant 1 × 10^5^conidia/mL. The mixed biofilm was incubated at 37°C for 24 h and processed by SEM.

## Abbreviations

EPS: Extracellular polysaccharides substance
ECM: Extracellular matrix
SEM: Scanning electron microscopy
TEM: Transmission electron microscopy
CLSM: Confocal laser scanning microscopy
PDA: Potato dextrose agar
SCV: Small colony variants
eDNA: Extracellular DNA
NETS: Neutrophil extracellular traps
FUN-1: Fluorescent vital dye FUN®-1 (2-chloro-4-[2,3-dihydro-3-methyl-{benzo-1,3-thiazol-2-yl}-methylidene]-1-phenylquinolinium iodide)
ConA: Concanvalin A
DAPI: Fluorescent dye, 6-diamidino-2-phenylindole
PI: Propidium iodide
CFU: Colony forming unit
